# Correction: High iodine adsorption performances under off-gas conditions by bismuth-modified ZnAl-LDH layered double hydroxide

**DOI:** 10.1039/d0ra90095h

**Published:** 2020-09-16

**Authors:** Trinh Dinh Dinh, Dongxiang Zhang, Vu Ngoc Tuan

**Affiliations:** School of Chemistry and Chemical Engineering, Beijing Institute of Technology Beijing 102488 China boris@bit.edu.cn +86 13366112230; Vilas 849, Quality Testing Lab, Center for Research and Development Science Technology Tien Nong Thanh Hoa 442410 Vietnam; Faculty of Electric-Electronic Engineering, Nam Dinh University of Technology Education Nam Dinh 420000 Vietnam

## Abstract

Correction for ‘High iodine adsorption performances under off-gas conditions by bismuth-modified ZnAl-LDH layered double hydroxide’ by Trinh Dinh Dinh *et al.*, *RSC Adv.*, 2020, **10**, 14360–14367, DOI: 10.1039/D0RA00501K.

The authors regret that the reference for Fig. 1 was omitted. The reference has been added below.

**Fig. 1:**
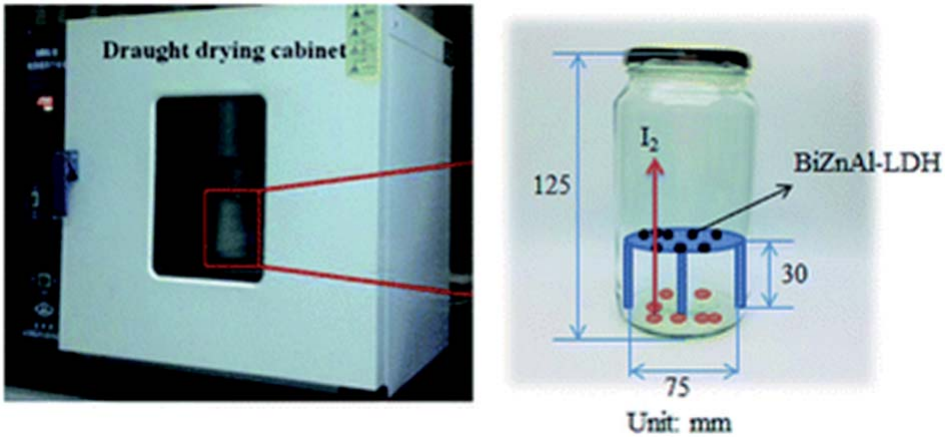
Schematic of the device for the iodine adsorption experiments in static air.^1^

The Royal Society of Chemistry apologises for these errors and any consequent inconvenience to authors and readers.

## Supplementary Material
